# Using Electromyography to Detect the Weightings of the Local Muscle Factors to the Increase of Perceived Exertion During Stepping Exercise

**DOI:** 10.3390/s8063643

**Published:** 2008-06-01

**Authors:** Ya-Ju Chang, Chin-Chih Liu, Cheng-Hsiang Lin, Peih-Ling Tsaih, Miao-Ju Hsu

**Affiliations:** 1 Physical Therapy Department and Graduate Institute of Rehabilitation Science, Chang Gung University, Tao-Yuan, Taiwan; E-mail: yjchang@mail.cgu.edu.tw; 2 Department of Physical Therapy, Shu-Zen College of Medicine and Management, Kaohsiung, Taiwan; 3 Department of Statistics, Tunghai University, Taichung, Taiwan; 4 Graduate Institute and School of Physical Therapy, College of Medicine, National Taiwan University, Taipei, Taiwan; 5 Faculty of Physical Therapy, College of Health Science, Kaohsiung Medical University, Kaohsiung, Taiwan; Department of Rehabilitation, Kaohsiung Medical University Hospital, Kaohsiung, Taiwan; E-mail: mjhsu@kmu.edu.tw

**Keywords:** Fatigue, stepping, electromyogram (EMG), heart rate, rating of perceived exertion, GEE model

## Abstract

Rate of perceived exertion (RPE) is a clinically convenient indicator for monitoring exercise intensity in cardiopulmonary rehabilitation. It might not be sensitive enough for clinicians to determine the patients' physiological status because its association with the cardiovascular system and local muscle factors is unknown. This study used the electromyographic sensor to detect the local muscle fatigue and stabilization of patella, and analyzed the relationship between various local muscle and cardiovascular factors and the increase of RPE during stepping exercise, a common exercise program provided in cardiopulmonary rehabilitation. Ten healthy adults (4 males and 6 females) participated in this study. Each subject used their right bare foot to step up onto a 23-cm-high step at a constant speed until the RPE score reached 20. The RPE, heart rate (HR), and surface EMG of the rectus femoris (RF), vastus medialis, and vastus lateralis were recorded at 1-minute intervals during the stepping exercise. The generalized estimating equations (GEE) analysis indicated that the increase in RPE significantly correlated with the increase in HR, and decrease in median frequency (MF) of the EMG power spectrum of the RF. Experimental results suggest that the increase in RPE during stepping exercise was influenced by the cardiovascular status, localized muscle fatigue in the lower extremities. The weighting of the local muscle factors was more than half of the weighting of the cardiovascular factor.

## 1. Introduction

Stepping exercise is widely used to increase or maintain physical fitness, as it is convenient, private, low cost, and requires no special motor skills. Research has demonstrated that stepping effectively improves cardiovascular fitness [9,22,34,38], decreases body fat [22], and increases lower limb strength [38].

Clinically, finding a sensitive and convenient indicator for exercise intensity is important to achieve optimal training effects from stepping exercise without inducing substantial fatigue. The Borg's rate of perceived exertion (RPE) consists of numbers ranging from 6–20 that individuals use to rate their levels of exertion. The RPE has been utilized as a subjective indicator of exercise intensity by both young adults [21] and aged persons [50]. The RPE gauges physical sensations a person experiencing during physical activity.

Studies have shown that RPE has a strong linear correlation with heart rate (HR) and aerobic power in healthy individuals [30] and patients with cardiac diseases [31]. However, in some cases, such as in sedentary subjects [54] or during isometric exercises [48], changes in RPE are not proportional to changes in HR. Carton et. al. argued that at low levels of exertion, muscle input is more important than central input in sensing efforts[12]. Since the RPE does not always have a strong linear relationship with HR, utilizing the RPE to monitor the patients' cardiopulmonary rehabilitation without knowing the weighting of the cardiovascular and other factors, such as local muscle factors might be dangerous.

Quadriceps femoris, which comprises the rectus femoris (RF), vastus medialis (VM), vastus lateralis (VL), and vastus intermedius, is a primary muscle group that is activated during stepping exercise. The RF is a two joint muscle whereas the VM and VL are single joint muscles. The balance between VM and VL is important in maintaining stability of the patellar during dynamic tasks [11,14,18,19,28,29,40,46,49]. Quadriceps femoris can be fatigued during stepping exercise. Therefore, physical effort may be increased as additional motor units are recruited to compensate for the fatigue-related force drop. Additionally, fatigue-related metabolic accumulation can stimulate mechanoreceptors and nociceptors through group III, IV afferents [15,26,27], thus inhibiting the spinal circuit, causing discomfort and increase in RPE.

Surface electromyography (EMG) is a non-invasive method of examining muscle function in vivo (e.g., activation level and fatigue). The median frequency of the EMG power spectrum has been demonstrated to be an effective indicator of fatigue as it is has been reported to be reduced during neuromuscular fatigue and is related to metabolic accumulation [3,5,6,7,16,17]. The intensity of EMG was reported to be associated with both fatigue and changes in muscle activation level [17]. This study utilized the EMG of RF, VM, and VL to monitor local muscle factors during stepping exercise.

Effort perception is a complicated notion. Establishing appropriate RPE weights for the contribution of HR and local muscle factors during stepping exercise is important for clinicians to determine a patient's physiological status and, thus, to appropriately implement a rehabilitation program. This study attempts to use the electromyographic sensor to detect the physiology status of the local muscle status and to determine appropriate weights for HR and local muscle factors for RPE during stepping exercise.

## 2. Methods

### 2.1. Participants

Ten (4 males and 6 females, aged 20–30 years old) young adults with no physical disability participated. Each subject provided informed consent. None of the subjects had previous history of neuromuscular or skeletal diseases.

### 2.2. Experimental Procedure

The EMG signals from the RF, VM, and VL were recorded using a bipolar surface electrode with a fixed interelectrode distance of 2 cm (B&L Engineering, Canada). Following skin abrasion with an alcohol-soaked cotton pad, electrodes were placed on the muscle bellies. For the RF, electrodes were placed at about 6 cm proximal to the superior border of the patella parallel with the long axis of the muscle. The VM and VL electrodes were located half-way between the muscle belly and the distal tendinous insertion of the respective muscle when the quadriceps were isometrically contracted and parallel to muscle fibers. The relative positions were based on those utilized in a previous study [19]. Electrodes were then secured with adhesive tape. The reference electrode was placed over the bony surface of the tibial bone. The EMG activity was on-site pre-amplified by a factor of 350 and further amplified at the mainframe amplifier. The mainframe amplifier had an input impedance >10 MΩ, a common mode rejection ratio of 100dB at 60Hz, and a gain range of 0.5–100,000 times (Gould Inc., Valley View, OH, USA). The EMG activity was monitored on an oscilloscope and digitized using a 12-bit resolution analog-to-digital converter (Metrabyte DAS 1600) at 1000 Hz.

Each subject sat on a chair with the knee joint flexed at 60° and performed two maximal voluntary contractions (MVCs), each for 5 seconds. The force and the EMG during MVCs were recorded for normalization purpose. The subject then used their right bare foot to step up onto a 23-cm-high step at a constant speed until the RPE score reached 20. Stepping speed was controlled with a metronome. During stepping, subjects wore an ankle-foot orthosis on their left foot to prevent plantar flexion and to ensure that all stepping effort was made by the right leg. Heart rate was monitored with a heart rate monitor (Polar-Electro, Kempele, Finland). The HR, and EMG of RF, VM, and VL were recorded continuously during stepping, whereas the RPE was reported by the subject at 1-minute intervals during stepping.

### 2.3. Data Analysis

The root-mean-square EMG (rEMG) amplitude of RF, VL, and VM were derived for the entire ascending phase of each step cycle and were normalized to that during MVCs. Although both VM and VL muscles are innervated by femoral nerve, the fiber type composition and the best activation knee angle are different. The change in the ratio of VM and VL (VM:VL ratio) may contribute to patella instability and knee pain, and thus possibly increase the RPE. Therefore, we also computed the ratio of VM and VL to represent the patella stabilization. This ratio was calculated by dividing the normalized rEMG of VM by that of VL. A fast Fourier transform was performed on the raw EMG of RF and the median frequency was calculated from the transformed signal. We did not calculate the median frequency of VM and VL because RF, VM, and VL are synergists in generating knee extensor torque. To simplify the model, we use only RF to represent the status of knee extensor torques generated. The HR, normalized rEMG of RF (EMG-RF), VM:VL ratio, and median frequencies (MF-RF) were applied for further statistical analyses.

Liang and Zeger (1986) and Zeger and Liang (1986) introduced generalized estimating equations (GEEs) to account for the correlation between observations in generalized linear regression models [37,55]. The GEE model is utilized in this study to determine the relationship between RPE and different independent variables. Since data are collected for the same individual across successive time points, these repeat observations are correlated over time. If this correlation is not considered, then the standard errors of the parameter estimates will be invalid and hypothesis testing results will be nonreplicable. GEE model can be described by Equation (1).

Let *Y_it_* are observations for subject *i* at time *t*,

(1) $$Y_{it}=\beta_{0}+\sum_{j=1}^{J}\beta_{1j}X_{\text{itj}}+\beta_{2}t+{ɛ}_{it}$$

*β*_0_ is the intercept, *X_ijt_* is the independent variable *j* for subject *i* at time *t*, *β*_1_*_j_* is the regression coefficient for independent variable *j*, *J* is the number of independent variables, *t* is time, *β*_2_ is the regression coefficient for time, and *ε_it_* is the ‘error’ for subject *i* at time *t* . Here we assume that observations on different subjects are independent, although we allow for association between outcomes observed for the same subject. If there is a quadratic linear trend between *Y_it_* and time, the GEE model can be expressed as

(2) $$Y_{it}=\beta_{0}+\sum_{j=1}^{J}\beta_{1j}X_{\text{itj}}+\beta_{2}t+\beta_{3}t^{2}+{ɛ}_{it}$$

## 3. Results and Discussion Experimental Section

**Figure 1.**
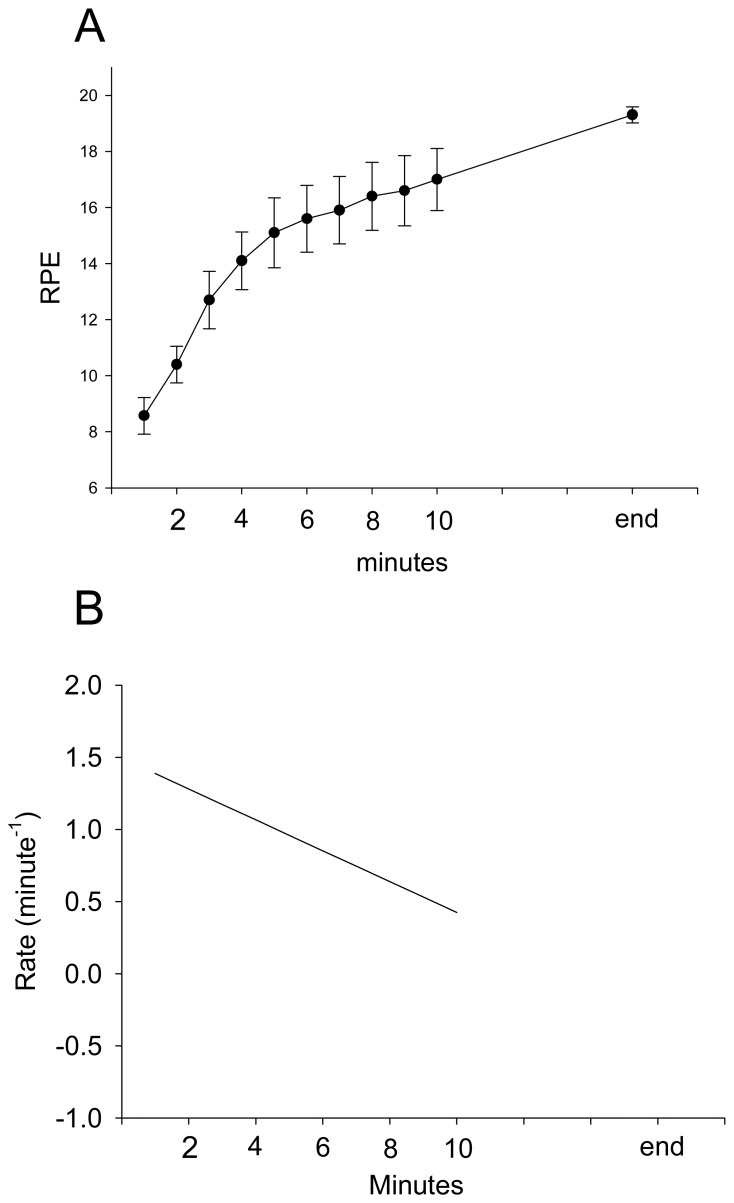
The group average and standard error of RPE during stepping exercise (A). The rate of RPE changes during stepping exercise (B).

**Figure 2.**
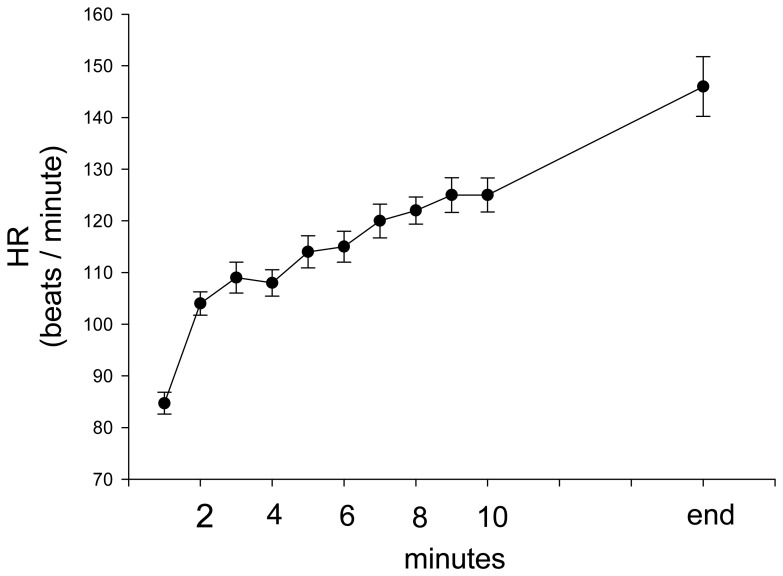
The group average and standard error of HR during stepping exercise.

**Figure 3.**
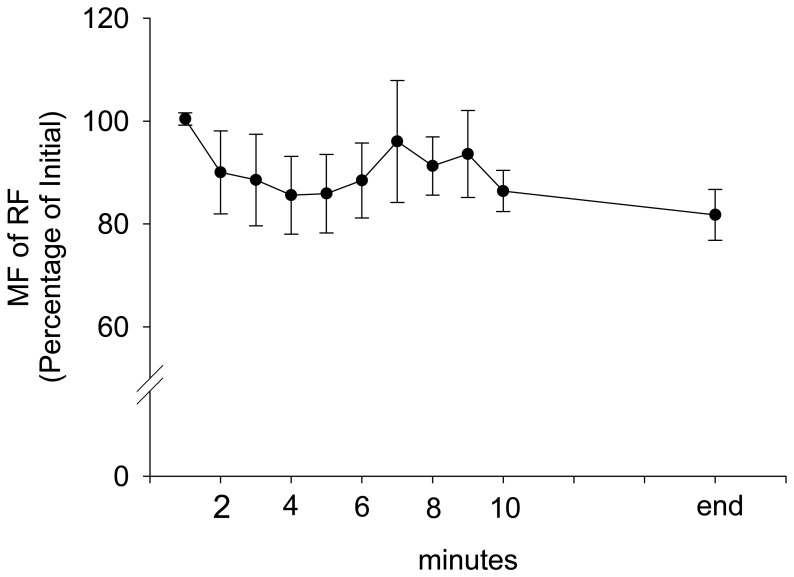
The group average and standard error of MF of RF during stepping exercise. The MF is represented as the percentage of the MF before stepping exercise.

**Figure 4.**
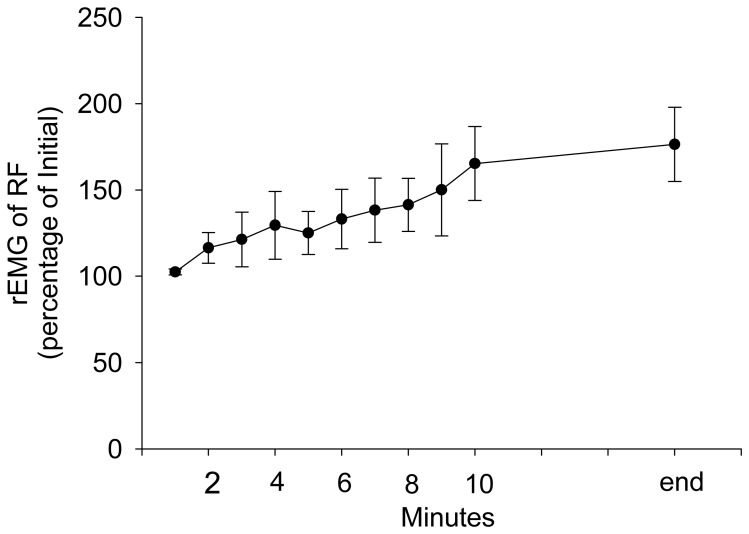
The group average and standard error of rEMG of RF during stepping exercise. The rEMG is represented as the percentage of the rEMG before stepping exercise.

**Figure 5.**
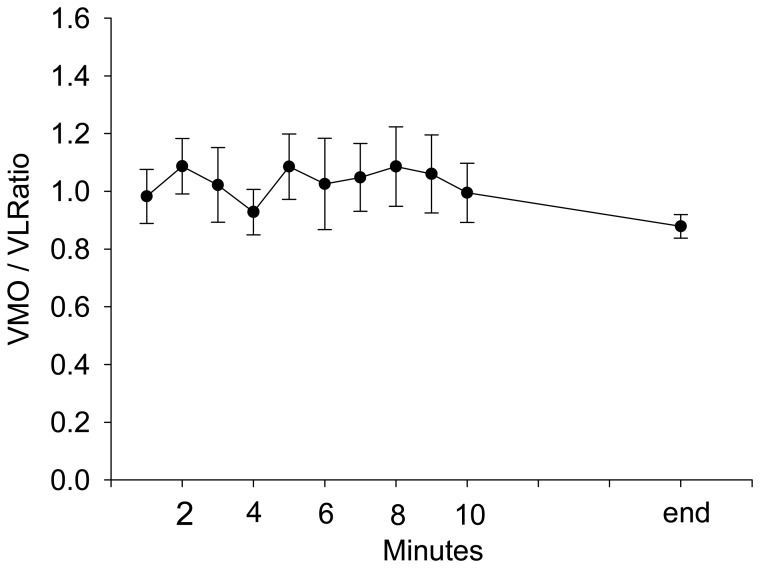
The group average and standard error of VMO / VL ratio during stepping exercise.

During stepping exercise, the HR, rEMG of RF, MF of RF, and VM:VL ratio showed different patterns during stepping. RPE and HR increased during stepping exercise (Figure 1A, 2). The MF of RF decreased initially and later was inconsistent (Figure 3). The rEMG had a tendency of increasing during stepping (Figure 4). The VM:VL ratio was initially close to 1.0 and was inconsistent near the end of the exercise (Figure 5) .

Table 1 presents the result of GEE analysis based on Equation (2). Time, Time^2^, MF of RF and HR were significant explanatory variables for RPE; specifically, each increase in 1 unit for HR and MF of RF led to an increase of 3.827 unit and resulted in a decrease of 2.6306 unit of RPE, respectively, after adjusting for time and other variables. However, rEMG and VM:VL ratio are insignificant explanatory variables for RPE (Table 1). Additionally, the analytical results indicate that a curve linear trend exists between RPE and time after adjusting for other variables. This implies [∂(RPE) / ∂Time) ] =-0.1174*Time+1.5629 which the rate change in RPE with respect to time is a linear function. The rate change of RPE is -0.1178 unit per minute (Figure 1B).

## 4. Discussion

The major finding of this study was that the increase in RPE during stepping exercise was related to both the cardiovascular status, HR, and the local muscle factors, including the MF of RF. Furthermore, as was the case with all other factors in the model, the rEMG of RF and VM:VL ratio were not significantly associated with RPE.

**Figure 6.**
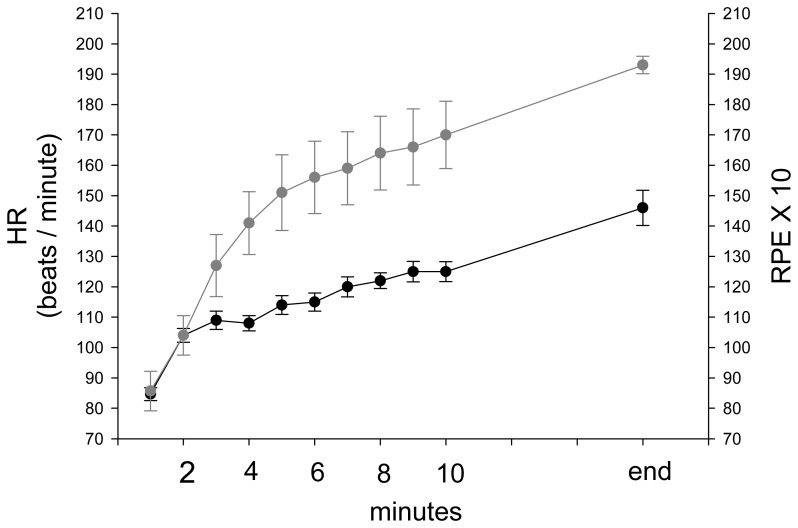
The group average and standard error of HR (black line) and RPEX10 (gray line) during stepping exercise.

Stamford demonstrated a linear relationship between RPE and HR during progressively increasing workloads and submaximal constant load, obtaining correlations ranging from r = 0.71 to 0.91 [52]. Similarly, in our study, as shown in the Table 1, the GEE model suggested a significant linear relationship between RPE and HR after adjusting for other variables. Borg suggested that a high correlation exists between a person's RPE × 10 and actual HR during physical activity and, thus, a person's RPE may provide a good estimate of actual HR during exercise. However, Garcin et al. assessed the relationship between RPE and HR under three loads during exhausting exercises, arguing that the equation HR= 10 × RPE was invalid for exercise at constant load until exhaustion [25]. In our study, the subjects stepped at the same rate until exhaustion. As seen in figure 6, HR and RPE × 10 were similar at the beginning (< 3 minutes) and the equation appeared to be appropriate. Nevertheless, after that, RPE × 10 separated from HR and became higher than HR throughout stepping exercise, indicating that the equation was invalid (Figure 6) during constant workload and supporting that weighting of other factors, such as local muscular factors, should be considered.

According to the GEE model in this study, the MF of RF was negatively correlated with RPE during stepping exercise when other factors were controlled. The reduction in median frequency of the EMG power spectrum has been typically considered as an indicator of fatigue as it has been noted during fatigue by maximum and submaximum voluntary contractions [4,8,16,17]. The shift in the EMG power spectrum is considered to be associated with motor units recruitment [7,8], fatigue-induced metabolic accumulation[26], change in intracellular PH [36], and reduction in muscle fiber conduction velocity [1,4,6,53]. In this study, decrease to MF during stepping exercise indicated that stepping exercise did produce local muscle fatigue. Increase of RPE might reflect the need for compensating the reduction of force generation caused by muscle fatigue. Furthermore, increased RPE is also likely needed to overcome discomfort and peripheral neural circuit inhibition arising from group III and IV afferent stimulated by fatigue-induced metabolic accumulation [26].

The averaged rEMG of RF showed a trend to increase during stepping (Figure 4), however, the GEE analysis results demonstrated that the rEMG of RF was not significantly correlated with the RPE when other factors were controlled. The amplitude of rEMG was associated with the sum of motor unit action potential [41] and the amplitude is likely affected by fatigue and recruitment. When fatigue is induced by submaximal task, the amplitude of EMG increases; this increase has been attributed to recruitment of additional motor units [2] and/or increased firing rates. Simultaneously, the amplitude of motor unit action potential of previously recruited motor units can decrease due to fatigue-related neuromuscular transmission failure [23,24]. As the change in rEMG during fatigue induced by submaximal exercise is complicated and stepping exercise in this study is submaximal, that the rEMG was not significant in the GEE model is not surprising. The VM and VL are believed to balance the patella during dynamic exercise. Some studies determined that the VM:VL ratio is correlated with the patella-femoral syndrome [10,40,42,46,51] and contraction of hip adductors [19,32,39], although results were contradictory [13,33,45,49,]. Changes in the VM:VL ratio during stepping exercise likely indicates that effort is required to balance the patella or change stepping strategies, such as activating hip adductors, and thus would increase the RPE. Change in VM:VL ratio is also likely associated with varying amounts of fatigue in VM and VL. Edgerton et al. reported [20] a greater proportion of type-I fiber in the vastus medialis compared that in the VL. However, other studies reported that the diameter of type-I fibers were not significantly different between VM and VL [35,44]. In our study, the VM:VL ratio was insignificantly related to RPE change when other factors were controlled, indicating that the the stabilization of the patella remained unchanged during stepping until exhaustion.

## 5. Conclusion and Clinical Application

This study used EMG sensors to detect muscle status and indicated that the increase to RPE during stepping exercise is affected by localized muscle factors in addition to the conventionally known cardiovascular exertion. The relationships among these contributing factors were determined by the GEE model. This study provides an approach using a model to differentiate and quantify contributory factors to exercise tolerance during stepping. Based on our model, the weighting of HR was more than that of the local muscle factor, suggesting that the cardiovascular response is the major limiting factor to exercise tolerance. However, the weighting of the local muscle factor was more than half of the weighting of the cardiovascular factor, indicating this factor is also important. With our model as a reference, we can test patient groups with the protocol used in this study to identify the limiting factors of exercise tolerance during stepping. This is especially useful when we attempt to determine the limiting factors for patients with comorbidities, such as coronary heart disease coexisted with neuromuscular disease. After establishing a model for the patient group, we can identify the primary factor limiting exercise performance and set up training regimen accordingly. For example, when the weighting of local muscle is less than half of HR, clinicians should focus on cardiopulmonary endurance training. In contrast, muscle strengthening/endurance training should be emphasized. In this way, we can set up clinical treatment guidelines for different patient groups.

The limitation is that this model might be unable to directly fit patient population. Future studies for establishing models for patients with cardiovascular diseases, neuromuscular diseases, and patellafemoral syndrome, etc are suggested.

## Figures and Tables

**Table 1.** Analysis of GEE Parameter Estimates.

Parameter	Estimate	Standard Error	95% Confidence Limits		Z	P
Intercept	6.0983	2.8901	0.4339	11.7627	2.11	0.0349
Time	1.5629	0.2134	1.1446	1.9813	7.32	<.0001
Time^2^	-0.0587	0.0132	-0.0847	-0.0328	-4.43	<.0001
rEMG of RF	-0.7789	0.9227	-2.5873	1.0296	-0.84	0.3986
MF of RF	-2.6306	1.0238	-4.6372	-0.6240	-2.57	0.0102
VM:VL Ratio	0.5146	0.6947	-0.8471	1.8763	0.74	0.4589
HR	3.8270	1.7014	0.4922	7.1617	2.25	0.0245
